# Resistance-Nodulation-Cell Division (RND) Transporter AcrD Confers Resistance to Egg White in *Salmonella enterica* Serovar Enteritidis

**DOI:** 10.3390/foods11010090

**Published:** 2021-12-30

**Authors:** Xiaojie Qin, Yanhong Liu, Xianming Shi

**Affiliations:** 1 School of Health Science and Engineering, University of Shanghai for Science and Technology, Shanghai 200093, China; qxj19900709@163.com; 2 Molecular Characterization of Foodborne Pathogens Research Unit, Eastern Regional Research Center, Agricultural Research Service, United States Department of Agriculture, Wyndmoor, PA 19038, USA; yanhong.liu@usda.gov; 3 School of Agriculture and Biology, Shanghai Jiao Tong University, Shanghai 200240, China

**Keywords:** *Salmonella* Enteritidis, egg white, AcrD, stress resistance, cell invasion

## Abstract

The excellent survival ability of *Salmonella enterica* serovar Enteritidis (*S*. Enteritidis) in egg white leads to outbreaks of salmonellosis frequently associated with eggs and egg products. Our previous proteomic study showed that the expression of multidrug efflux RND transporter AcrD in *S*. Enteritidis was significantly up-regulated (4.06-fold) in response to an egg white environment. In this study, the potential role of AcrD in the resistance of *S*. Enteritidis to egg white was explored by gene deletion, survival ability test, morphological observation, Caco-2 cell adhesion and invasion. It was found that deletion of *acrD* had no apparent effect on the growth of *S*. Enteritidis in Luria-Bertani (LB) broth but resulted in a significant (*p* < 0.05) decrease in resistance of *S*. Enteritidis to egg white and a small number of cell lysis. Compared to the wild type, a 2-log population reduction was noticed in the *ΔacrD* mutant with different initial concentrations after incubation with egg white for 3 days. Furthermore, no significant difference (*p* > 0.05) in the adhesion and invasion was found between the wild type and *ΔacrD* mutant in LB broth and egg white, but the invasion ability of the *ΔacrD* mutant in egg white was significantly (*p* < 0.05) lower than that in LB broth. This indicates that *acrD* is involved in virulence in *Salmonella*. Taken together, these results reveal the importance of AcrD on the resistance of *S*. Enteritidis to egg white.

## 1. Introduction

Eggs are an important and integral part of the human diet. These are consumed all over the world and possess natural physical and chemical defenses to prevent the contamination of microorganisms [1]. Egg white, as a chemical barrier, is generally a hostile environment for bacterial survival and growth because of its unfavorable conditions, such as alkaline pH, nutritional limitations and antibacterial molecules [2,3]. However, the risk of *Salmonella* contamination is a serious threat to human health as well as egg production and processing. In particular, *Salmonella enterica* serovar Enteritidis (*S*. Enteritidis) represents the predominant serotype that is involved in food-borne diseases due to the consumption of eggs and egg products. More importantly, *S*. Enteritidis presents an exceptional ability to survive in egg white in contrast to other *Salmonella* serotypes [4,5,6].

It is important to understand the resistance mechanisms of *S*. Enteritidis to egg white. Previous workers have revealed key information through the use of molecular biological techniques such as site-directed mutagenesis, transposon-mediated insertional mutagenesis, in vivo expression and DNA arrays at the transcriptional level, which may be helpful in explaining the underlying survival mechanism in this foodborne pathogen [7,8,9]. While genes identified in those studies were mainly involved in iron transport, biotin synthesis, energy metabolism, cell envelope maintenance, DNA synthesis and repair, motility and pathogenicity. Furthermore, genes such as outer membrane channel-related gene *tolC* [10], DNA repair-related gene *yafD* [7] and stress response-related genes *uspAB* [11] were identified by mutagenic analysis and were considered as main players for the survival of *S*. Enteritidis in egg white. On the other hand, according to our previous study, the transcriptomic and proteomic profiles of *S*. Enteritidis exposed to egg white were analyzed using RNA-Seq and iTRAQ analysis to reveal potential important metabolic pathways, such as stress response, iron acquisition, amino acid and biotin synthesis, transport and regulation [6,12]. A highly up-regulated expression (4.06-fold) of the stress response related protein AcrD was found in *S*. Enteritidis in response to whole egg white at the protein level [12].

Gram-negative bacteria such as *Salmonella* usually have multidrug efflux transporters, which have been found to recognize and excrete various structurally unrelated compounds from the cell. Among the multidrug efflux pumps, members of the RND (Resistance-Nodulation-cell Division) family appear to be the most effective efflux systems in those bacteria. *Salmonella* has five RND-type efflux systems: AcrAB, AcrAD, AcrEF, MdtABC and MdsABC [13]. The RND transporter AcrD has a unique biological role, which can remove antimicrobial compounds, such as aminoglycosides, from the bacterial cell. Inactivation of *acrD* resulted in changes in the expression of 403 genes involved in basic metabolism, stress responses and virulence [14]. Furthermore, the deletion of *acrD* led to a significant reduction in biofilm formation and down-regulated expression of key biofilm formation-related proteins encoded by *csgBD* [15]. Previously, the deletion of *acrD* resulted in an increased sensitivity to antibiotics, dyes and detergents in *S.* Typhimurium [16,17]. Furthermore, AcrD also contributes to copper and zinc resistance in *Salmonella* [18]. Previous works have demonstrated that *Salmonella* usually infects the human host through the ingestion of contaminated food products. This bacterium is able to resist the adverse environment of the gastrointestinal tract and then adhere, colonize and invade host intestinal epithelial cells, leading to human infections and diseases [19,20]. To our knowledge, the role of *acrD* in *S*. Enteritidis resistance to antibacterial egg white is not yet clear.

Hence, this study aimed to uncover the role of *acrD* in the resistance and virulence of *S*. Enteritidis to egg white by gene expression analysis, gene deletion, survival ability test, cellular morphology analysis, Caco-2 cell adhesion and invasion assays. These results will provide new information to help elucidate the resistance mechanisms of *S*. Enteritidis to egg white.

## 2. Materials and Methods

### 2.1. Bacterial Strains

*S*. Enteritidis strain SJTUF10978, isolated from chicken wings, was used as the wild-type (WT) strain in this study. *Escherichia coli* DH5α and *Salmonella* MRL0026 were utilized as reference strains for cell adhesion and invasion assays. These strains were stored at −80 °C in LB (Luria-Bertani) broth, including 50% (*v*/*v*) glycerol. All strains were propagated overnight at 37 °C on LB agar before experiments.

### 2.2. Caco-2 Cell Culture Preparation

The human colon adenocarcinoma cell line Caco-2, obtained from Shanghai Fuheng Biotechnology Co., Ltd. (Shanghai, China) (FH0029), were routinely maintained in DMEM (Dulbecco’s Modified Eagle’s Medium, Gibco, Pittsburgh, PA, USA) medium containing 1% non-essential amino acids (Coolaber, Beijing, China), 10% fetal bovine serum (Fuheng biology, Shanghai, China), 100 U/mL penicillin (Hyclone, Shanghai, China) and 100 μg/mL streptomycin (Hyclone, Shanghai, China) at 37 °C with 5% CO_2_. Meanwhile, cells were sub-cultured every 2–3 days and used between passages 5 and 10.

### 2.3. Egg White and Its Filtrate Preparation

SPF (Specific Pathogen Free) eggs used in this study were purchased from Boehringer Ingelheim Vital Biotechnology Co., Ltd. (Beijing, China). Fifty eggs were stored in a 37 °C incubator with 65% RH (Relative Humidity) for 5 days as required for every independent biological repeat. Egg white was collected by homogenization and centrifugation as previously described [12]. Egg white filtrate (FEW, less than 10 kDa) was acquired by centrifugation using ultrafiltration tubes with the cut-off limit of 30 kDa and 10 kDa according to our previous method [21].

### 2.4. Gene Expression Analysis

Total RNA from log-phase cells of *S*. Enteritidis in whole egg white and LB broth was extracted using Trizol reagent (Invitrogen, Carlsbad, CA, USA) following the manufacturer’s instructions. The RNA concentrations were determined using a NanoDrop 2000c spectrophotometer (Thermo scientific, South Logan, UT, USA), and the quality of RNA was evaluated using 1% agarose gel electrophoresis. Furthermore, genomic DNA treatment and cDNA synthesis was conducted using the PrimeScript RT reagent kit following the manufacturer’s instructions (TaKaRa, Dalian, China). The gene expression of *acrD* was tested by RT-qPCR analysis (Eppendorf, Hamburg, Germany) as previously described [12], using primer pair of *acrD*-F (5′-ACGCAACAGCAGACCC-3′) and *acrD*-R (5′-GCCCAGACCGCTAATT-3′). The relative expression of *acrD* in *S*. Enteritidis was calculated by the comparative cycle threshold method [22]. For data normalization, 16S rRNA was utilized as a reference gene.

### 2.5. Construction of acrD Deletion Mutant Strain

In-frame deletion of *acrD* was generated based on the previously described homologous recombination knockout method [23]. The primers used are shown in Table 1. In addition, strains and plasmids utilized for the deletion are listed in Table 2. Firstly, the fragment of homologous arms (i.e., upper arm and lower arm) was amplified from the genomic DNA of wild-type *S*. Enteritidis SJTUF10978 by overlap extension PCR. Secondly, this fragment was cloned into the pMD_19_-T plasmid carrying an ampicillin resistance gene to produce pMD_19_-*ΔacrD*. The correct pMD_19_-*ΔacrD* plasmid was digested with *Sac* I and *Xba* I and then ligated into the pRE112 plasmid carrying a chloramphenicol resistance gene and a sucrose-sensitive gene. Then, the obtaining pRE112-*ΔacrD* plasmid was imported into *E. coli* SM10 *λpir* using CaCl_2_ transformation method. The recombinant plasmid was then extracted and transformed into the *S*. Enteritidis wild-type strain by electroporation to obtain a single-crossover strain. The resulting strain was induced by 8%(*w/v*) sucrose to finish a second crossover. Finally, suspected colonies were chosen and confirmed by DNA sequencing and PCR analysis to acquire the *acrD* deletion mutant (*ΔacrD*).

### 2.6. Measurement of Bacterial Growth

Overnight cultures of *S*. Enteritidis wild-type and *ΔacrD* strains in LB broth were collected by centrifugation and diluted to the cell density of OD_600_ ≈ 0.1. Then, cultures were incubated at 37 °C with continuous shaking at 200 rpm. The bacterial growth curve was measured at regular time (1 h) intervals by a Bioscreen C Analyzer (OY Growth Curves, Finland).

### 2.7. Survival Ability of S. Enteritidis Strains in Egg White and Its Filtrate

The survival of *S*. Enteritidis wild-type and *ΔacrD* strains in egg white and its filtrate was measured according to our previously described method [12]. Bacteria (1 mL) at logarithmic phase were collected, washed twice using sterile PBS (Phosphate-Buffered Saline, 1.8 mM KH_2_PO_4_, 10 mM Na_2_HPO_4_, 2.7 mM KCl, 137 mM NaCl, pH 7.2) and suspended in PBS. The bacterial suspension was adjusted to approximately 1 × 10^7^ CFU/mL and 1 × 10^4^ CFU/mL by dilution in PBS. Then in a 96-well microplate, 20 μL aliquots of the bacterial suspensions were inoculated into 180 μL of egg white and 180 μL of its filtrate, respectively. It was mixed to give a final concentration of 1 × 10^6^ CFU/mL and 1 × 10^3^ CFU/mL, respectively. The above mixtures were incubated at 37 °C for 24 h. Viable bacteria after incubation were enumerated by plating 100 μL of the treated cell suspensions on LB agar and incubated at 37 °C for 24 h.

### 2.8. Scanning Electron Microscopy (SEM) Analysis

The cell morphology of *S*. Enteritidis wild-type and *ΔacrD* strains in LB broth, egg white and egg white filtrate at 37 °C for 1 day was observed using a Sirion 200 SEM (FEI Company, Hillsboro, OR, USA) as previously described [21].

### 2.9. Adhesion and Invasion Assays

The adhesive and invasive ability of *Salmonella* strains and *E. coli* DH5α were investigated according to a previous method [26] with some modifications. The 48-h, 80% confluent Caco-2 monolayers were sub-cultured and placed into a 12-well plate at a density of approximately 1 × 10^5^ cells/well. Bacterial strains were inoculated in LB broth overnight at 37 °C. Bacterial cells were recovered by centrifugation at 13,800× *g* for 5 min, washed twice using DEME medium and suspended in DEME medium to a final concentration of 10^7^ CFU/mL. Then, bacterial suspensions and Caco-2 cells were mixed at a ratio of 100:1 and then incubated for 1 h at 37 °C in a 5% CO_2_ incubator. The unattached bacteria after incubation were removed after incubation by washing with PBS. 1% Triton X-100 was added to release the attached bacteria at 37 °C for 5 min. Then, the suspensions were serially diluted, and 20 μL of each dilution was plated on LB agar and then incubated at 37 °C for 24 h. Counted colonies were recorded as the total adhesive bacterial population. The adhesion rate of bacteria was represented as the ratio of the number of adhesive bacteria compared to that of initial inoculated bacteria.

Similarly, in the invasion test, infected Caco-2 cells were incubated in a DMEM medium containing 1% penicillin and streptomycin for 1 h at 37 °C to kill extracellular bacteria. Serial dilutions of the lysates were plated on LB agar to enumerate invading bacterial populations. The invasion rate of bacteria to Caco-2 cells was represented as the ratio of the number of invading bacteria compared to that of cell-adhesion bacteria.

### 2.10. Statistical Analysis

Three independent experiments were conducted in all assays, and each treatment was carried out in triplicate. Data were evaluated via one-way ANOVA using SAS software. Duncan’s multiple range test (*p* < 0.05, *p* < 0.001) was used to identify the difference in survival, cell adhesion and invasion ability between wild-type *S*. Enteritidis and the mutant.

## 3. Results and Discussion

### 3.1. Expression of acrD in S. Enteritidis in Response to Whole Egg White and Construction of acrD Mutant

**Figure 1:**
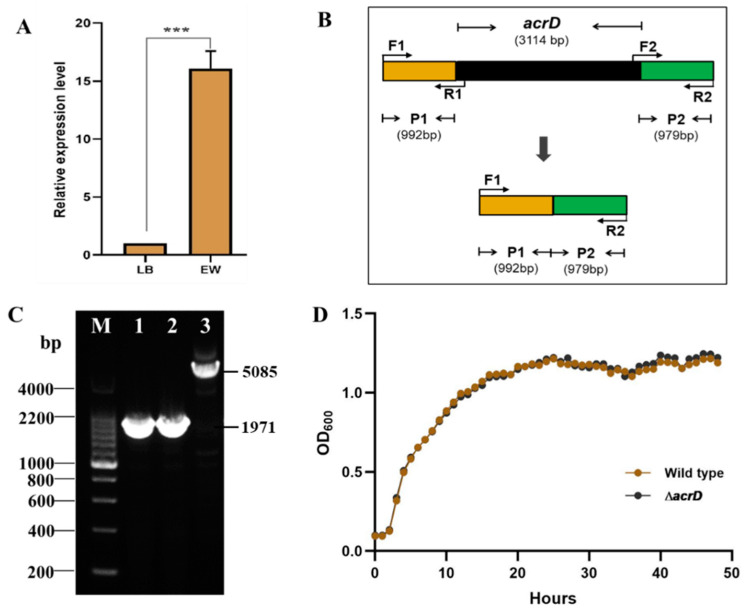
(**A**) Relative expression level of *acrD* of S. Enteritidis in whole egg white (EW) compared to that of Luria-Bertani (LB) broth at the mRNA level. Vertical bars show standard deviation. Asterisk indicates statistical differences according to Duncan’s multiple range test at *p* < 0.001 (***) level. (**B**) The in-frame deletion of *acrD*. P1: upstream fragment, P2: downstream fragment. (**C**) Confirmation of the successful construction of *ΔacrD* mutant by PCR using F1 and R2 primers. M: DNA marker, 1/2: *ΔacrD* mutant, 3: wild type. (**D**) The growth curve of S. Enteritidis wild type and *ΔacrD* mutant in LB broth.

In previous studies, up-regulated expression of *acrD* at the mRNA level has been demonstrated in *S*. Enteritidis in response to low concentrations of egg white, e.g., 10% egg white and 80% egg white [6,9]. To test whether *acrD* was up-regulated in the whole egg white (i.e., 100% egg white), the expression of *acrD* in *S*. Enteritidis exposed to the whole egg white was further analyzed using RT-qPCR in this study. As shown in Figure 1A, the expression of *acrD* was significantly (*p* < 0.001) up-regulated (16.09-fold) in whole egg white compared with that in LB broth at the mRNA level. This gene expression data at the mRNA level was consistent with the proteomic data, which also showed up-regulated expression of AcrD in *S*. Enteritidis exposed to whole egg white at the protein level [12]. Hence, these results suggest that *acrD* may play a potential role in the resistance of *S*. Enteritidis to egg white.

*Salmonella* can resist adverse conditions through gene expression regulation. For example, two universal stress-related genes, *uspA* and *uspB,* of *S*. Enteritidis 147^str^ are highly expressed in egg white, and a decreased colonization ability was observed to the magnum and isthmus of the oviduct when these genes were deleted [11]. The promoter of out membrane channel gene *tolC* was activated by egg white at 42 °C, and mutagenic analysis showed that *tolC* had an important role in *S*. Enteritidis survival in egg white [10]. Furthermore, the specific gene SEN1393 results in higher survivability of *S*. Enteritidis in egg white [27]. Although the *acrD* gene was expressed under different environmental stress conditions (e.g., antibiotics, detergents, metal) and seemed to contribute to stress resistance [15,16,18,20], there is no concrete evidence on the functional role of *acrD* in *S*. Enteritidis under egg white stress.

To better understand the role of *acrD* (encoding 1037 amino acids, a multidrug efflux RND transporter) in the resistance of *S*. Enteritidis to egg white, this gene (3114 bp) was deleted successfully in *S*. Enteritidis strain SJTUF10978 to obtain a *ΔacrD* deletion mutant (Figure 1B,C). The in-frame deletion mutant was confirmed by PCR and DNA sequencing (Figure 1B,C). Moreover, similar growth patterns of wild-type and *ΔacrD* strains in LB broth were found at 37 °C (Figure 1D), indicating that *acrD* is not required for *S.* Enteritidis growth in LB broth.

### 3.2. Survival Study of S. Enteritidis ΔacrD Mutant in Egg White and Its Filtrate

**Figure 2:**
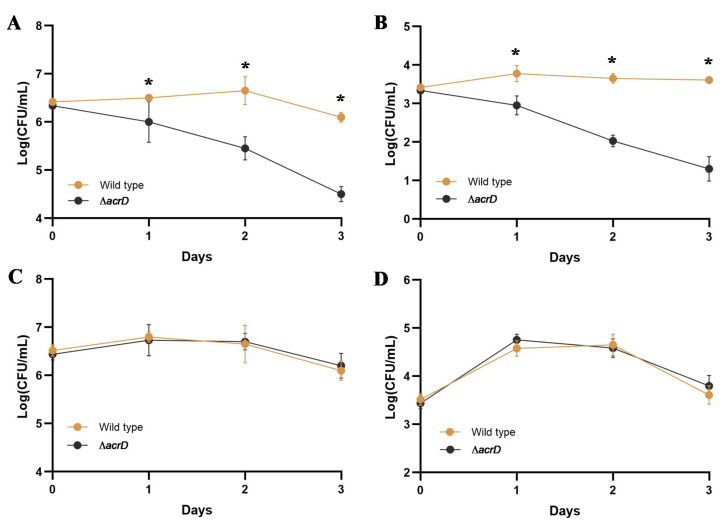
Survival ability of *S*. Enteritidis wild type and *ΔacrD* mutant in whole egg white (**A**,**B**) and its filtrate (**C**,**D**). Survival of *S*. Enteritidis in whole egg white with initial concentrations of 10^6^ CFU/mL (**A**) and 10^3^ CFU/mL (**B**). Survival of *S*. Enteritidis in egg white filtrate with initial concentrations of 10^6^ CFU/mL (**C**) and 10^3^ CFU/mL (**D**). Three independent experiments were performed, and the results of representative experiments were shown. Error bars indicate the standard deviation of three replicates. Asterisks (*) indicate significant differences in the survival ability between wild type and *ΔacrD* mutants (*p* < 0.05).

To explore the role of *acrD* in the survival of *S*. Enteritidis in egg white, the wild type and *ΔacrD* mutant were exposed to egg white and surviving bacteria were enumerated via plate counts on LB agar. As shown in Figure 2, the survival ability of *ΔacrD* mutant was significantly lower than that of the wild type in egg white (*p* < 0.05) (Figure 2A,B). More importantly, a 2-log population reduction was observed for the *ΔacrD* mutant after incubation in egg white for 3 days with different initial concentrations (i.e., 10^3^ and 10^6^ CFU/mL) (Figure 2A,B). These results demonstrate that *acrD* confers resistance to egg white in *S.* Enteritidis.

Generally, egg white filtrate (FEW, less than 10 kDa) has been used as a food matrix to reveal the antibacterial activity of egg white proteins [10,28]. Therefore, to explore the role of *acrD* in the resistance of *S.* Enteritidis to antibacterial egg white proteins in the present study, the wild-type strain and its *ΔacrD* mutant were exposed to egg white (containing various types of proteins) and egg white filtrate without the main antibacterial proteins. As shown in Figure 2C,D, the loss of *acrD* had no significant (*p* > 0.05) effect on the resistance of *S.* Enteritidis in egg white filtrate regardless of the initial cell concentrations, indicating that *acrD* plays a critical role in *S.* Enteritidis resistance to egg white proteins.

The RND transporter AcrD has a unique biological role in multidrug resistance, which can remove antimicrobial drugs such as aminoglycosides from the bacterial cell, and the RND family requires interaction with outer membrane channel TolC to function [13,29]. In addition, a previous study has demonstrated that RND transporters, such as AcrD, are necessary for the secretion of enterobactin, which chelates iron to enable bacterial growth under iron-limiting conditions [30]. Meanwhile, TolC has an important role in the resistance of *S.* Enteritidis to egg white ovotransferrin at 42 °C [10]. These results suggest that AcrD may contribute to *Salmonella* iron homeostasis to resist ovotransferrin in egg white. Antibacterial component experiments are needed to further confirm this hypothesis.

### 3.3. Cellular Morphology of S. Enteritidis in Egg White under SEM

**Figure 3:**
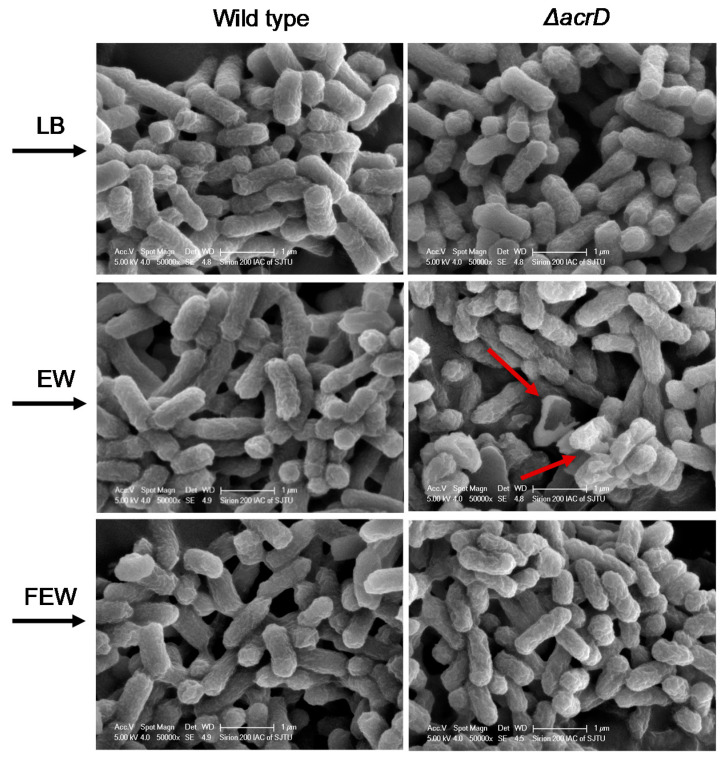
SEM micrographs of *S*. Enteritidis wild type and *ΔacrD* mutants in LB broth, egg white (EW) and egg white filtrate (FEW) at 37 °C for 1 day. Red arrows highlight clear examples of morphology changes. Magnification = 50,000×, bar marker = 1 μm.

The cellular morphology of *S.* Enteritidis strains (wild type and *ΔacrD* mutant) in LB broth, egg white and egg white filtrate at 37 °C for 1 day was observed by SEM. As shown in Figure 3, no significant morphological change was found between WT and *ΔacrD* in LB broth. However, a small number of *ΔacrD* cells exposed to egg white were lysed, compared with that of the WT. In contrast, there was no apparent morphological difference between WT and *ΔacrD* in egg white filtrate.

It has been commonly suggested that antibacterial proteins and peptides (e.g., lysozyme, ovotransferrin, defensins) are the main antibacterial factors of egg white that prevent bacterial growth, and the bacterial cell membrane is the main target of these antibacterial components [3,31]. For example, the bactericidal mechanisms of egg white lysozyme are mainly involved in hydrolyzing the β-1,4 glycosidic bonds of bacterial peptidoglycan, whereas the peptidoglycan layer is a key shape determining factor of the bacterial cell membrane [32,33]. Cationic peptides produced by the degradation of lysozyme and ovotransferrin, as well as other antibacterial peptides from egg white such as β-defensins, could interact electrostatically with negative charges on the outer membrane (e.g., anionic phospholipids, lipopolysaccharides and lipoteichoic acid) of bacterial cells, leading to bacterial death due to the leakage of substances [34]. Hence, in combination with the results of survival ability of the *S*. Enteritidis WT and its *ΔacrD* mutant in egg white filtrate, we speculated that damaged cells of *ΔacrD* are caused by antibacterial components in egg white.

### 3.4. The Adhesion and Invasion Ability of S. Enteritidis to Caco-2 Cells

**Figure 4:**
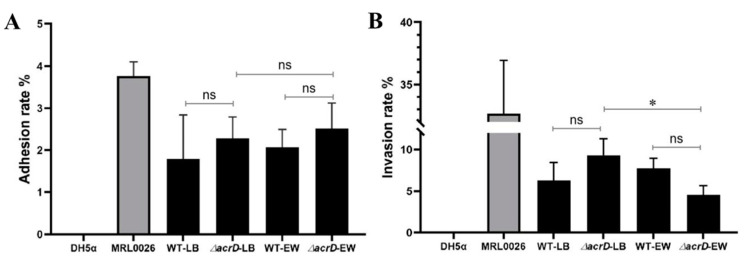
Adhesion (**A**) and invasion (**B**) ability of Caco-2 cells by *S.* Enteritidis wild type (WT), *ΔacrD* mutant and control strains in egg white (EW) and LB broth at 37 °C. Non-adherent/invasive *E. coli* DH5α and highly invasive *Salmonella* MRL0026 were used as negative and positive controls, respectively. Mean values and standard deviations were calculated from three replicates. Asterisk (*) indicates that there is a significant difference between the invasion rate of *ΔacrD* in egg white and that in LB broth (*p* < 0.05). ns, no significant difference.

Cell adhesive and invasive ability are usually used to evaluate the potential virulence of bacteria [31,35,36]. In this study, the adhesion and invasion abilities of Caco-2 cells by the *S*. Enteritidis wild type and its *ΔacrD* mutant were further investigated in LB broth and egg white. Non-adherent/invasive *E. coli* DH5α and the highly invasive *Salmonella* MRL0026 strain were used as negative and positive controls, respectively. The results showed that no significant difference (*p* > 0.05) in the adhesion rate was observed between the wild type and *ΔacrD* mutant in LB broth or egg white (Figure 4), indicating that the loss of *acrD* had no significant effect on the adhesion ability of *S*. Enteritidis. Similarly, the invasion rate of the *ΔacrD* mutant was basically consistent with that of the wild type in LB and egg white (*p* > 0.05). However, the invasion rate of the *ΔacrD* mutant in egg white (4.54%) was significantly (*p* < 0.05) lower than that in LB broth (9.30%) (Figure 4). These results indicate that the invasion ability of *S*. Enteritidis was influenced by egg white for cells lacking *acrD*.

Previous studies have confirmed that AcrD is related to the virulence of bacteria. For example, the infected ability of *Salmonella* was significantly reduced in its ability to infect INT 407 cells when either AcrD, AcrB or AcrF were missing [37]. Inactivation of *acrD* resulted in changes in the expression of some virulence-related genes [14]. Although no significant differences (*p* > 0.05) in the adhesion and invasion rates between the wild-type and *ΔacrD* mutant in LB broth or egg white were found in this study, there was a significant difference (*p* < 0.05) between the invasion rate of the *ΔacrD* mutant when in egg white versus LB broth (Figure 4). Combined with the other authors’ findings, the results of this study indicated that *acrD* is involved in virulence in *Salmonella* in response to egg white; however, the extent of this role requires further investigation.

## 4. Conclusions

This study revealed that AcrD conferred resistance to egg white in *S*. Enteritidis strain SJTUF10978 by analyzing an *acrD* deletion mutant. Meanwhile, this protein appears to be involved in virulence in *S*. Enteritidis in response to egg white. These findings broaden the understanding of the RND protein related to efflux pumps that mediates the resistance of *Salmonella* in egg white. Collectively, this study provides some novel insights into the resistance mechanism of *S*. Enteritidis to egg white.

## Figures and Tables

**Table 1:** Primers used for *ΔacrD* mutant construction.

Primer	Sequence (5′ to 3′)
*acrD*-F1	GCTCTAGACTCTACGCCGCTGCTGA (*Xba* I)
*acrD*-R1	**GGCCGGGAGCTAAAGGGGAA**CCTCGTGTTT
*acrD*-F2	**TTCCCCTTTAGCTCCCGGCCA**GCCTGATAC
*acrD*-R2	CGAGCTCGGCGACGAATAAGTTGCTGTG (*Sac* I)

The 20-bp overlap sequences for amplification of the fragments of homologous arms is shown in bold. Restriction enzyme sites are underlined.

**Table 2:** Strains and plasmids used in this study.

Strains or Plasmids	Relevant Characteristics	Reference or Source
*S*. Enteritidis SJTUF10978	Wild-type strain	Lab stock
*Δ* *acrD*	*acrD* deletion mutant of *S*. Enteritidis SJTUF10978	This study
*E. coli* DH5α	Host for cloning	Lab stock
*E. coli* SM10 (*λpir*)	*thi thr*-1 *leu*6 *pro*A2 *his*-4 *arg* E2 *lac*Y1 *gal*K2, *ara*14*xyl*5 *sup*E44, *λpir*	[24]
pMD_19_-T	Cloning vector, Amp^r^	TaKaRa, China
pMD_19_-*ΔacrD*	pMD_19_-T containing a 3113 bp *acrD* deletion PCR product	This study
pRE112	pGP704 suicide plasmid, *pir* dependent, *ori*T *ori*V *sac*B, Cm^r^	[25]
pRE112-*ΔacrD*	pRE112 containing a 3113 bp *acrD* deletion PCR product	This study

## Data Availability

All data related to the research are presented in the article.
